# Supplementary material for: Males of Dalbulus maidis Attract Females Through Volatile Compounds with Potential Pheromone Function: A Tool for Pest Management
Source: Insects. 2025 Oct 2;16(10):1021. doi: 10.3390/insects16101021 (PMC12565353; doi:10.3390/insects16101021)
Supplement: Supplementary file 1 [file insects-16-01021-s001.zip › insects-3823474-supplementary.pdf]

Supplementary Material Sanches *et al.* 2025

Males of *Dalbulus maidis* attract females through volatile com-pounds with potential pheromone function: a tool for pest management

Insects (MDPI)

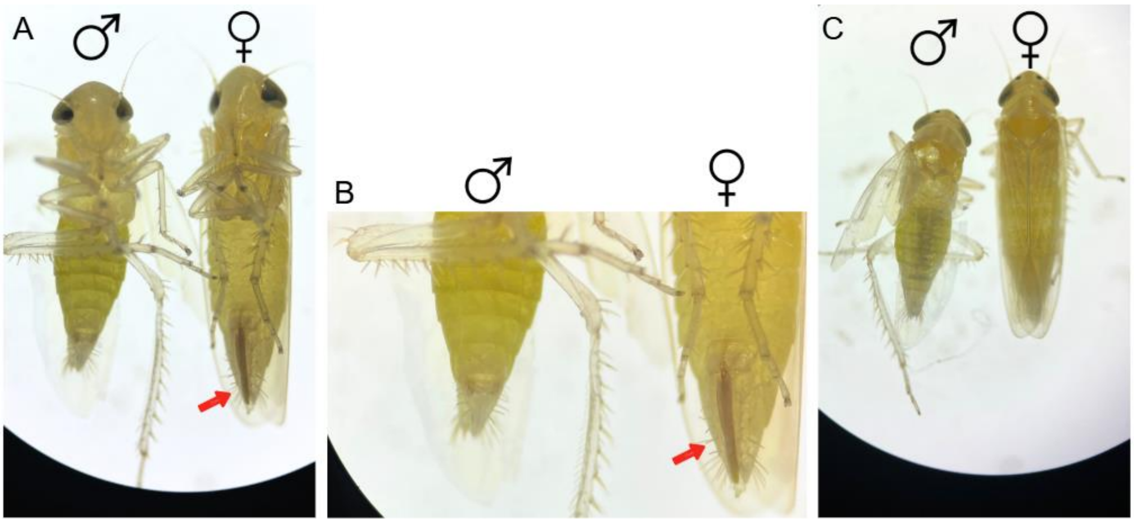

Figure S1. Male and female of *Dalbulus maidis*. **(a) -** Ventral view of male and female, respectively. **(b)** - Terminal abdomen of male and female. **(c)** - Dorsal view of male and female. Arrows indicate the ovipositor of the female.

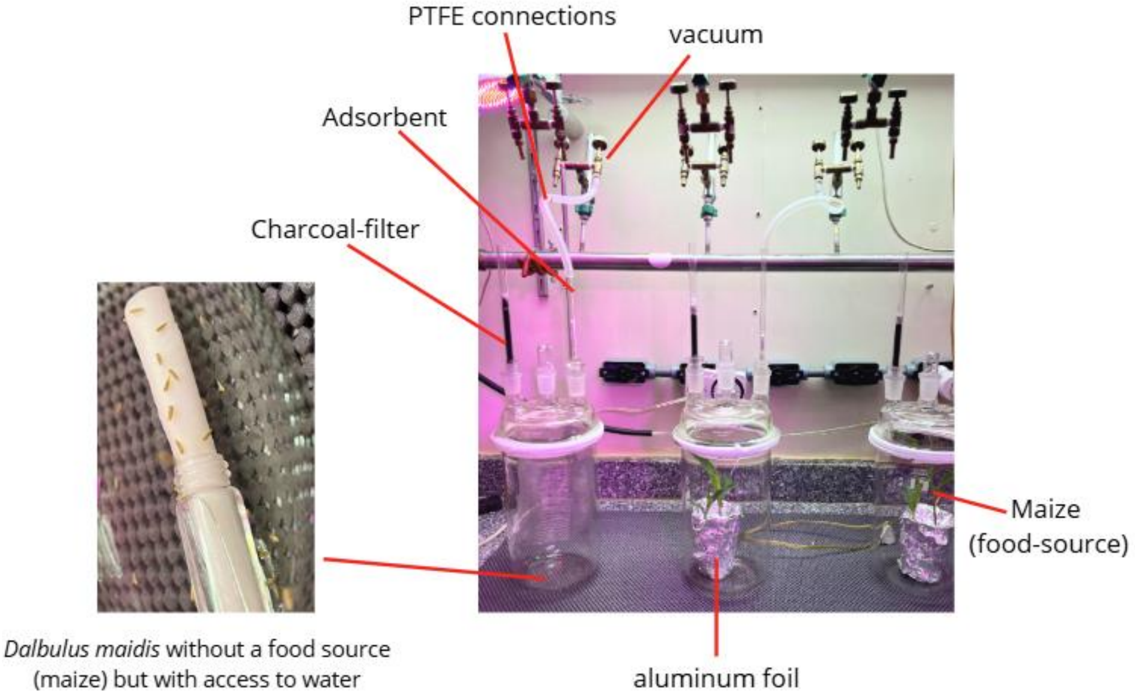

Figure S2. Volatile sampling system for *Dalbulus maidis* in the presence and absence of its food source (maize).
